# Impact of transfer from pediatric gastroenterology to adult gastroenterology care in eosinophilic esophagitis

**DOI:** 10.1093/dote/doaf012

**Published:** 2025-02-27

**Authors:** Katherine F Webber, James C Slaughter, Dhyanesh A Patel, Girish Hiremath

**Affiliations:** Department of Medicine, Division of Gastroenterology, Hepatology and Nutrition, Vanderbilt University Medical Center, Nashville, TN, USA; Department of Biostatistics, Vanderbilt University Medical Center, Nashville, TN, USA; Department of Medicine, Division of Gastroenterology, Hepatology and Nutrition, Vanderbilt University Medical Center, Nashville, TN, USA; Department of Pediatrics, Division of Pediatric Gastroenterology, Hepatology and Nutrition, Vanderbilt University Medical Center, Nashville, TN, USA

**Keywords:** eosinophilic esophagitis, loss to follow-up, medication non-adherence, outcome research, transfer of care

## Abstract

Given the chronic and progressive course of eosinophilic esophagitis (EoE), patients with pediatric-onset EoE will require uninterrupted gastroenterology (GI) care as they reach adulthood. Yet, the effectiveness of transferring and integrating EoE patients from pediatric GI (pGI) to adult GI (aGI) care has not been studied. To address this gap, we assessed loss to follow-up, duration from the last pGI to the first aGI encounters (clinic visit and EGD), and its impact on clinical course and medication non-adherence in EoE patients. We identified 58 EoE patients who initially received pGI care and were transferred to aGI between 2017 and 2023 within our institution’s shared electronic medical record environment. Demographic, clinical, endoscopic, and histologic data were analyzed using descriptive statistics, survival analysis, Cox regression models, and paired comparisons. Loss to follow-up was 16%. The median duration from the last pGI clinic visit to the first aGI clinic visit was 299 days, and that for the last pGI EGD to the first aGI EGD was 730 days. A significantly higher odds of heartburn (McNemar *P*-value = 0.01) and higher medication non-adherence rates (7% vs. 26%) were noted in 49 patients who established care with the aGI. The endoscopic and histologic severity remained unchanged. In EoE patients, transferring from pGI to aGI care is associated with loss of follow-up, deterioration of symptoms, and medication non-adherence. There is a critical need to develop optimized protocols to ensure a seamless transfer of care for EoE patients.

## BACKGROUND

Eosinophilic esophagitis (EoE) is an increasingly prevalent, chronic, and progressive immunoinflammatory disorder of the esophagus.^1^ Affected children commonly present with feeding difficulties, food aversion, vomiting, and abdominal pain, whereas teenagers and adults frequently present with heartburn, dysphagia, and esophageal food impaction. Although various options (including dietary elimination and pharmacologic therapies) are available to manage EoE, currently, there is no cure for this clinicopathologic condition.^2^^,^^3^ Since it can begin in childhood and continue into adulthood, an increasingly larger cohort of pediatric EoE patients are expected to age into adulthood and transition their care from a pediatric to an adult healthcare model.

Healthcare transition (HCT) is the process of changing from a pediatric to an adult healthcare model with or without transfer to a new clinician.^4^ The core elements of HCT include discussing transition policy, tracking progress, assessing skills, developing an HCT plan, transferring to adult-centered care and integration into adult practice, confirming transfer completion, and eliciting consumer feedback.^5^ Previous studies have investigated transition policy, tracking progress, assessing skills, and eliciting consumer feedback in EoE patients.^6–8^ However, very little research has focused on transferring from pediatric to adult-centered care and integration into adult practice, one of the critical phases of successful HCT. This represents a significant knowledge gap as loss of follow-up, clinical deterioration, and medication non-adherence have been reported during this period in other chronic and progressive conditions, such as inflammatory bowel disease (IBD).^9^

To address this critical knowledge gap, we aimed to determine the loss of follow-up among pediatric EoE patients who transferred from pediatric gastroenterology (pGI) to adult GI (aGI) care within our institution. Our secondary aims were to assess the duration between the last pGI and the first aGI encounters (including the clinic visits and EGD) and its association with the patient’s features. Additionally, in a subset of patients with longitudinal follow-up, we explored the impact of the transfer period on their clinical status, medication non-adherence, and endoscopic and histologic features.

Since we were studying the effectiveness of the transfer of care of EoE patients within our institution’s shared electronic medical record (EMR) environment, we hypothesized that there would be minimal (10%) loss to follow-up and specific patient and clinical features would influence the duration between pGI and aGI encounters. Furthermore, given the progressive nature of EoE, we hypothesized that the clinical status, medication non-adherence, and endoscopic and histologic features would worsen during the transfer period.

## METHODS

### Study design and subjects

In this retrospective study, using the SlicerDicer function, a self-service reporting that is intuitive and customizable, in our institution’s shared EMR (EPIC), we identified 58 patients diagnosed with EoE who received pGI care between ages 0 and 19 with a subsequent visit to the Vanderbilt Digestive Disease Center (Adult Gastroenterology) between 2017 and 2023. The shared EMR system was made available for clinical documentation in our institution in 2017. Like many other academic centers, our institution did not offer formal preparatory transition services, and we transferred patients from pGI to aGI at around 18 years of age.

EoE was defined per the existing diagnostic guidelines.^10^^,^^11^ Specifically, children with symptoms of esophageal dysfunction and a peak eosinophil count (PEC) of ≥15 eosinophils per high-power field (eos/hpf) were classified as active EoE and those with a known diagnosis of EoE and PEC of <15 eos/hpf were considered to have inactive EoE. Children (≤18 years) diagnosed with EoE but with inadequate documentation that their EoE care was being transferred to the aGI were excluded. The study was approved by the Vanderbilt Institutional Review Board (# 230645).

### Variables collected and analyzed

From the pGI records, we gathered demographic (sex, race, insurance), clinical (age at EoE diagnosis, duration of symptoms before EoE diagnosis, presenting symptoms), allergic comorbidities (self-reported food allergies, eczema, asthma), medication exposures (proton pump inhibitors [PPIs], topical steroids [TSs], biologics [such as dupilumab]), endoscopy (number of esophagogastroduodenoscopies [EGDs] under the care of pGI, date of last pediatric EGD, at the time of last pediatric EGD was the patient symptomatic or asymptomatic, EoE endoscopic reference scores [EREFS]^12^), and histologic (PEC)^13^ data.

The following information was collected from the medical records of the patients who established care with the aGI: date of first (or establishing care) aGI clinic visit, symptoms, medications at the time of establishing care, and date of first adult EGD, and the first adult EREFS and PEC. The EGDs were performed at the discretion of the GI overseeing the patient’s care, and the severity of the esophageal abnormalities was scored per the EREFS. The histologic evaluation of the esophageal biopsies during pGI care was performed by a pediatric pathologist and, during aGI care, was conducted by an adult pathologist.

### Statistical analysis

Descriptive statistics were used to describe the cohort. The categorical variables are presented as percentages [*n* (%)], and the continuous variables are presented as a median and interquartile range (median [QR]) and mean and standard deviation (mean $\pm$ SD).

EoE patients who did not establish care with the aGI at our institution after being counseled by the pGI provider were classified as ‘lost to follow-up’. Survival analysis estimated the duration (in days) from the last pGI clinic visit and EGD to the first aGI clinic visit and EGD, respectively. Subjects were considered at risk from their last pGI visit if they met inclusion criteria. They were followed until their first aGI clinic visit, or 18 months passed without transferring to an aGI clinic or being censored before 18 months. Likewise, subjects were considered at risk from their last pGI EGD if they met inclusion criteria. They were followed until the date of their first aGI EGD, or 24 months passed without an aGI EGD, or censored before 24 months. Subjects were censored on 1/31/24 if they had not transferred to the aGI clinic and their most recent pediatric clinic visit was within 18 months of data extraction and if they had not had an aGI EGD and their most recent pGI EGD was within 24 months of data extraction. Based on the practical considerations (wait time to schedule an aGI clinic visit and aGI EGD) and our institutional policy (establishing care before performing elective EGD), we considered censoring at 18 months for the first aGI clinic visit and 24 months for the first aGI EGD would be sufficient to observe relevant outcomes (or trends) in our population. We assumed the censored subjects were comparable to those still at risk for transfer to the adult clinic at the time of censoring (independent censoring).^14^ Cox regression models were used to estimate the univariate and adjusted hazard ratios to examine the impact of patient and clinical features on the time from the last pGI clinic visit to the first aGI clinic visit and the time since the last pGI EGD to the first aGI EGD.

Finally, we explored the evolution of clinical, endoscopic, and histologic features across the transfer period among those who established aGI care. Given the intra-individual correlation (last pGI vs. first aGI), we used the McNemar test for paired comparisons of categorical variables. Where data were sparse, we presented only the proportions without conducting statistical tests. We also calculated the paired mean differences (95% confidence interval [CI]) between the last pGI and the first aGI EREFS and PEC, respectively. To ensure the robustness of our results, 5000 bootstrap samples were generated, and the confidence interval was bias-corrected and accelerated. All analyses were performed on R version 4.3.3, and a *P*-value of <0.05 was considered statistically significant.

## RESULTS

### Cohort characteristics

Our cohort predominantly comprised males (81%) and White patients (93%) with private insurance (91%). The duration of symptoms before receiving an EoE diagnosis was 12 (1–36) (median [IQR]) months, and the age at EoE diagnosis was 12 (8–15 years). Dysphagia (67%), abdominal pain (40%), heartburn (33%), and vomiting (31%) were among the most common presenting symptoms. Food allergy was noted in 47% of subjects. The median number of pGI EGDs was 5 (3–9) (Table 1).

**Table 1:** Characteristics of the cohort

		***N* = 58**
Male^*^		47 (81)
Race^*^	African American/Black	3 (5)
	White	54 (93)
	Others	1 (2)
Insurance^*^	Private	53 (91)
	Government	5 (9)
Duration of symptoms prior to EoE diagnosis (months)^#^		12 (1–36)
Age at EoE diagnosis (years)^†^		12 (8–15)
Symptoms at presentation to pGI^*^	Dysphagia	39 (67)
	Abdominal pain	23 (40)
	Heartburn	19 (33)
	Vomiting	18 (31)
	Food impaction	10 (17)
	Chest pain	9 (16)
	Nausea	6 (10)
	Failure to thrive	8 (14)
	Weight loss	3 (5)
Allergic comorbidities^*^	Food allergy	27 (47)
	Eczema	12 (21)
	Asthma	11 (19)
Number of EGDs under pGI care^†^		5 (3–9)
	1–4^*^	26 (45)
	5–9^*^	32 (55)

^*^ *n* (%);

^†^Median (interquartile range).

### Loss of follow-up and the interval between pGI and aGI encounters

**Fig 1:**
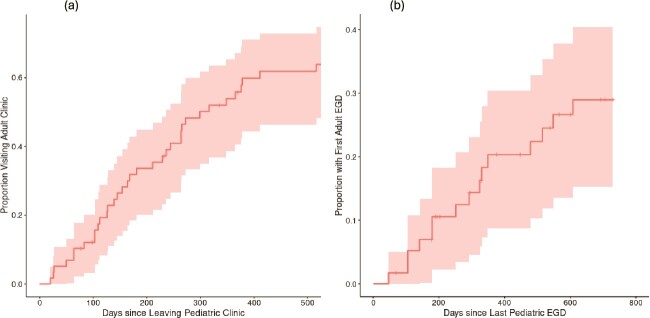
The overall survival curve showing duration (in days) from (a) last pGI clinic visit to the first aGI clinic visit and (b) last pGI EGD to the first aGI EGD.

Forty-nine (84%) out of 58 patients established care with the aGI, resulting in a loss to follow-up of 16%. The most common reasons for loss to follow-up included: scheduled aGI visit but did not show up or canceled multiple times [*n* = 5 (56%)], did not schedule aGI clinic visit [*n* = 3 (33%)], moved out of town or unable to locate [*n* = 3 (33%)], and incompatible insurance coverage [*n* = 1 (1%)]. The median interval from the last pGI clinic visit to the first aGI clinic visit was 299 days, and the last pGI EGD to the first aGI EGD (*n* = 27) was 730 days (Fig. 1). Cox regression models indicated that the duration from the last pGI encounters (clinic visit and EGD) to the first aGI encounters (clinic visit and EGD) were not associated with the age at diagnosis, number of pGI EGDs, and EREFS and PEC at the last pGI EGD.

### The transfer phase is associated with clinical deterioration and medication non-adherence

Next, we examined the change in the clinical status, medication non-adherence, and endoscopic and histologic features during the transfer of care period (Table 2). From the last pGI clinic visit to the first aGI clinic visit (*n* = 49), there was a notable decrease in the proportion of asymptomatic patients (57% vs. 43%) and an increase in the patients complaining of heartburn (5% vs. 20%). While more patients switched from asymptomatic status to symptomatic status than symptomatic status to asymptomatic status, this difference did not achieve statistical significance (McNemar *P*-value = 0.08). However, the odds of switching from not having heartburn at the time of the last pGI clinic visit to having heartburn at the time of the first aGI clinic visit was nine times higher than the odds of switching from having heartburn at the last pGI clinic visit to not having heartburn during the first aGI clinic visit. This observed difference was statistically significant (McNemar *P*-value = 0.01). The medication non-adherence rates increased from the last pGI clinic visit to the first aGI clinic visit (3% vs. 29%), particularly among those on a PPI and TS combination therapy (24% vs. 52%). However, the McNemar test could not be performed due to sparse data. In a subset of patients (*n* = 27) with the last pGI EGD and the first aGI EGD, the paired mean differences of EREFS [0.44 (−0.40 to 1.25); *P*-value = 0.44] and PEC [−5.76 (−30 to 13.67); *P*-value = 0.57] were comparable.

**Table 2:** Clinical features of the individuals who transferred from pediatric GI and established care with adult GI

		Last pediatric GI encounter (*N* = 58)	First adult GI encounter (*N* = 49)
Presentation*	Asymptomatic	33 (57)	21 (43)^†^
Symptoms*	Dysphagia	19 (33)	14 (29)
	Heartburn	3 (5)	10 (20)^‡^
	Vomiting	7 (12)	6 (12)
	Abdominal pain	5 (9)	5 (10)
Medications*	Non-compliance to medications	2 (3)	14 (29)
	PPI only	20 (35)	15 (31)
	TS only	2 (3)	3 (6)
	PPI + TS	30 (52)	12 (24)
	Dupilumab	4 (7)	5 (10)

^*^ *n* (%); EREFS, endoscopic reference score; PEC, peak eosinophil count per high power field; PPI, proton-pump inhibitor; TS, topical steroids.

^†^McNemar *P*-value = 0.08.

^‡^McNemar *P*-value = 0.01.

During the transfer interval, one (2%) patient presented to the emergency room with esophageal food impaction. While this patient underwent an urgent EGD by an aGI to remove the impacted food bolus, esophageal biopsies were not obtained during the procedure.

## DISCUSSION

Transfer of care remains a substantially understudied aspect of EoE.^6^ Given its chronic and progressive course and lack of curative therapy, individuals diagnosed with EoE in their childhood are expected to require continued care as they transition into adulthood. In this single-center descriptive study, we assessed the effectiveness of transferring and integrating EoE patients from pGI to aGI care within a shared EMR environment.

We observed that 16% of EoE patients were lost to follow-up. Studies involving IBD patients report up to 30% loss to aGI follow-up.^15^^,^^16^ Although the rate of loss to follow-up in our study was lower than the loss to follow-up rates reported in IBD patients, it exceeded our anticipated *a priori* threshold of 10%. It is conceivable that the loss to follow-up rates in EoE patients could be higher in non-academic settings and centers without a shared EMR environment.

The duration between the last pGI encounters (clinic and EGD) and the first aGI encounters (clinic visit and EGD) were 299 and 730 days, respectively. While there are no benchmarks to compare our data, like many other academic institutions, the average wait times for our aGI practice (clinic visits and procedures including EGD) are variable. They can be related to the patient status (new vs. return), insurance coverage, diagnosis, acuity of symptoms, and availability of specialized care (such as EoE clinic, IBD clinic, and celiac clinic).

Interestingly, our longitudinal follow-up revealed that the transfer phase was associated with deterioration in the clinical status and an increase in medication non-adherence, particularly to the PPI and TS combination therapy, perhaps due to the time-consuming and burdensome nature of preparing and administering multiple medications. It will be essential to investigate if decreasing the time from the last pGI encounters (clinic visit and EGD) and the first aGI encounters (clinic visit and EGD) can reduce the loss to follow-up rates, minimize deterioration of clinical status, and positively impact medication adherence, as shown in the IBD patient population.^17^

Taken together, loss to follow-up, worsening of the underlying condition, and suboptimal compliance to medications are some of the critical challenges associated with the transfer and integration phase of HCT, especially in adolescents and young adults with chronic diseases, including EoE.^18^^,^^19^ These challenges can be linked to the young age at diagnosis, lack of transfer readiness, incomplete understanding of the course of the disease, heavy medication burden coupled with a perceived lack of benefit of medications, nuances of the insurance coverage, risk-taking behavior, and higher rates of psychiatric comorbidities (such as depression, anxiety, and lack of social coping skills).^9^ Our results highlight the need to establish benchmarks specific to the transfer and integration phase of HCT (such as early introduction of transfer of care concepts, education sessions focused on the transfer of care, simplifying therapy when possible, a combined transition clinic when feasible, peer support groups or mentorship programs, and educating parents/guardians about fostering independence and self-management in their children) to ensure seamless transfer and the continuity of care for young adults with EoE. Proposed actionable steps to optimize the transfer of care from pGI to aGI are summarized in Supplementary Table 1.

The transfer approach described in our study aligns with the unstructured healthcare transfer model. This approach is known to be associated with a lapse in medical care,^20^ medication non-adherence,^21^ medical complications,^22^ and higher utilization of emergency and hospital services.^23^ Emerging data support the use of a structured healthcare transfer for patients with chronic illnesses to improve adherence to care, medication adherence, and decreasing disease-related complications and improve the sense of health-related self-efficacy, patient-initiated communication, and patient satisfaction when they participate in a structured transfer process.^24^^,^^25^ Such guidelines have already been published for adolescents with chronic conditions such as IBD^26^^,^^27^ and asthma.^28^^,^^29^ Developing and implementing structured healthcare transfer guidelines for EoE is imperative.

Our study has limitations. It is a single-center retrospective study conducted in an academic setting within a shared EMR environment. As such, it is prone to the limitations and biases associated with the study design. A significant limitation is that we lacked a suitable comparison group against which to contrast our results. Specific diseases (such as IBD), which have their unique requirements for clinic visits and endoscopic procedures, may not serve as appropriate control groups. Moreover, the absence of previously published data on this topic precludes us from comparing our results. Our results may not be generalizable to other practice settings, centers with a formal transition of care preparatory program, and those without a shared EMR. Although we had a modest sample size, the homogeneous characteristics limited our ability to conduct an in-depth evaluation of the impact of sex, race, and insurance on our outcomes of interest. While we analyzed data from patients who successfully transferred to adult GI care, this may not represent those lost to follow-up. Lastly, differences in the real-world clinical practice between pGI and aGI (including variations in wait times for the first available appointment, clinical, medication prescriptions [number of refills], and endoscopic and histologic assessments) and pathologists, even within an institution (academic and non-academic settings), may introduce variability and biases.

Despite these limitations, this study has several strengths. This is the first study to examine the effectiveness of the transfer from pGI to aGI in individuals with EoE. We leveraged the shared EMR environment and used SlicerDicer to ensure a systematic and standardized approach to identify eligible subjects and collect data, contributing to the robustness of the study methodology. While a multidisciplinary approach is ideal for managing EoE patients, the focus of this study was on GI care as GIs provide outpatient consultation, perform EGDs to assess the severity of their esophageal mucosal involvement, and obtain esophageal biopsies for histologic assessment. Finally, we employed robust statistical methods to analyze the data comprehensively.

In summary, this study provides original insights into the impact of the transfer from pGI to aGI care on the loss of follow-up, clinical status, medication non-adherence, and endoscopic and histologic features in EoE patients. By delving into the effectiveness of transferring care, an understudied area, the study identifies gaps and opportunities to optimize the care of EoE patients with a focus on collaboration between pGI and aGI providers. Furthermore, it highlights the urgent unmet need to establish benchmarks to ensure continuity of care for young adults with pediatric-onset EoE. A multi-institutional prospective study is warranted to address this critical unmet need.

## Supplementary Material

Supplementary_Table_1-01_30_25_doaf012

## ABBREVIATIONS

aGI, adult gastroenterology; EGD, esophagogastroduodenoscopy, EoE, eosinophilic esophagitis; EMR, electronic medical record; EREFS, endoscopic reference scoring system; GI, gastroenterology; HCT, healthcare transition; IBD, inflammatory bowel disease; PEC, peak eosinophil count; pGI, pediatric gastroenterology; PPI, proton-pump inhibitor; TS, topical steroids

## Notes

Funding Support: G.H. is supported by the National Institutes of Health (NIH) 1K23DK131341.

Conflict of Interest or Disclosures: G.H. serves as an advisor to Sanofi, Regeneron, and Takeda. Other authors declare no relevant conflicts of interest or disclosures.

Specific author contributions: K.F.W: Contributed to data curation, methodology, original draft preparation, reviewing, and editing. J.C.S: Contributed to formal analysis, methodology, visualization, reviewing, and editing the manuscript. D.A.P: Contributed to conceptualization, methodology, supervision, and reviewing and editing the manuscript. G.H: Contributed to conceptualization, methodology, supervision, and reviewing and editing the manuscript. Katherine Webber (Data curation, Methodology, Writing—original draft, Writing—review & editing), James Slaughter (Data curation, Formal analysis, Methodology, Visualization, Writing—review & editing), Dyanesh Patel (Conceptualization, Data curation, Investigation, Methodology, Supervision, Writing—review & editing), and Girish Hiremath (Conceptualization, Data curation, Formal analysis, Investigation, Methodology, Supervision, Visualization, Writing—review & editing).
