# Supplementary material for: Impact of transfer from pediatric gastroenterology to adult gastroenterology care in eosinophilic esophagitis
Source: Dis Esophagus. 2025 Feb 27;38(1):doaf012. doi: 10.1093/dote/doaf012 (PMC11878569; doi:10.1093/dote/doaf012)
Supplement: Supplementary_Table_1-01_30_25_doaf012 [file supplementary_table_1-01_30_25_doaf012.docx]

**Supplementary Table 1:** Proposed actionable steps to optimize the transfer of care of eosinophilic esophagitis (EoE) patients from pediatric to adult gastroenterology care.

| **Stage (Age)** | **Key Actions** | **Goals** | **Stakeholders** |
| --- | --- | --- | --- |
| Early preparation (12-15 years) | -Introduce the concept of transfer of care during routine visits and endoscopy  -Assess patients understanding of EoE, including triggers and management  -Educate on the basics of disease pathophysiology (such as allergen mediated, triggered by food and aero-allergens, medication use, compliance to therapy, endoscopic monitoring) | -Build patient’s comfort level with understanding and managing the disease  -Lay the foundation for independent care | -Pediatric provider, patient, parents/ caregivers |
| Transition planning (15-17 years) | -Develop an individualized transition plan  -Gradually encourage taking ownership of managing the disease (dietary restrictions, compliance to medications, etc)  -Assess readiness to transfer care to adult providers  -Educate about the differences between pediatric and adult care systems | -Ensuring that the patient understands the importance of managing EoE independently  -Identify barriers to a successful transfer of care | -Pediatric provider, patient, parents/ caregivers |
| Provider communication (16-18 years) | -Establish communication between pediatric and adult providers  -Share detailed medical history (including trigger foods, clinical presentation, endoscopic findings, biopsy results, medication history, compliance to therapy, treatment response, and potential barriers such as insurance) | -Facilitate continuity of care by ensuring the adult provider is adequately informed about the patients’ history and the course of EoE | -Pediatric and adult provider, patient (with or without parents/ caregivers) |
| Handoff care (17-19 years) | -If feasible, conduct a combined pediatric and adult provider transition clinic  -Introduce the adult care team  -Address psychosocial concerns (e.g., anxiety about adult care, adherence, challenges) | -Build trust with the adult provider  -Encourage long term self-management and adherence | -Adult provider, patient, support team (e.g., psychosocial support) |
| Post-transfer care (18+ years) | -Ensure that the patient has established care with the adult provider, monitor adherence to therapy and endoscopic evaluations  -Provide ongoing education on managing flares, monitoring symptoms  -Address barriers or psychosocial concerns | -Ensure that the patient is integrated and comfortable in adult care setting  -Set patient up for success in long-term self-management of EoE | -Adult provider, patient, support team (e.g., psychosocial support) |
